# Molecular signatures of age-associated chronic degeneration of shoulder muscles

**DOI:** 10.18632/oncotarget.7382

**Published:** 2016-02-14

**Authors:** Yotam Raz, Jan Ferdinand Henseler, Arjen Kolk, Zuotian Tatum, Niels Kuipers Groosjohan, Nisha E. Verwey, Wibowo Arindrarto, Szymon M. Kielbasa, Jochem Nagels, Peter A. C. 't Hoen, Rob G. H. H. Nelissen, Vered Raz

**Affiliations:** ^1^ Department of Orthopaedics, Leiden University Medical Center, Leiden, The Netherlands; ^2^ Department of Human Genetics, Leiden University Medical Center, Leiden, The Netherlands; ^3^ Department of Medical Statistics and Bioinformatics, Leiden University Medical Center, Leiden, The Netherlands

**Keywords:** shoulder disease, atrophy, fatty infiltration, muscle satellite cells, deep RNA-seq, Gerotarget

## Abstract

Chronic muscle diseases are highly prevalent in the elderly causing severe mobility limitations, pain and frailty. The intrinsic molecular mechanisms are poorly understood due to multifactorial causes, slow progression with age and variations between individuals. Understanding the underlying molecular mechanisms could lead to new treatment options which are currently limited. Shoulder complaints are highly common in the elderly, and therefore, muscles of the shoulder's rotator cuff could be considered as a model for chronic age-associated muscle degeneration. Diseased shoulder muscles were characterized by muscle atrophy and fatty infiltration compared with unaffected shoulder muscles. We confirmed fatty infiltration using histochemical analysis. Additionally, fibrosis and loss of contractile myosin expression were found in diseased muscles. Most cellular features, including proliferation rate, apoptosis and cell senescence, remained unchanged and genome-wide molecular signatures were predominantly similar between diseased and intact muscles. However, we found down-regulation of a small subset of muscle function genes, and up-regulation of extracellular region genes. Myogenesis was defected in muscle cell culture from diseased muscles but was restored by elevating MyoD levels. We suggest that impaired muscle functionality in a specific environment of thickened extra-cellular matrix is crucial for the development of chronic age-associated muscle degeneration.

## INTRODUCTION

Aging-associated muscle degeneration leads to functional impairments of daily activities of the elderly due to immobility and frailty. Muscle degeneration is prominent in neuromuscular disorders [1] and acute muscle disuse conditions [2]. In these conditions discovery of the associated molecular mechanisms have led to better understanding of the pathophysiological processes of these disorders [3]. However, the molecular changes occurring in muscle degeneration in chronic conditions are still not fully understood. This is partly due to multifactorial etiologies, slow progression with aging, variation between individuals and unavailability of suitable models. Shoulder complaints are common in the general population [4-6], and their prevalence progresses during aging to more than 40% [7, 8]. Disease in the shoulder's rotator cuff (RC) muscles is characterized by disability of the shoulder joint, leading to pain and functional impairment in daily activities. The four RC muscles function as intricate motor units that dynamically stabilize the shoulder joint and provide arm mobility [9, 10]. Initially, studies focused on the tendon degeneration [11-15], but recently muscle degeneration has been suggested to be primarily involved in affected RC muscles [16, 17]. Importantly, muscle degeneration in the shoulder is considered as a clinical determinant for surgical and non-surgical treatment outcomes [16, 18-20]. However, variable and non-sustained outcomes call for better understanding of the intricate balance of shoulder pathology and functionality [21, 22].

Within the spectrum of chronic muscle disease tearing of the shoulder's RC is common. In RC tears the supraspinatus muscle (SSp) is initially affected and as the disease progresses, the subscapularis muscle (SSc) can tear as well [23]. In chronic RC diseases the deltoid muscle (DM) is clinically not affected, and can be considered a reference muscle [17]. Previously, we showed that while atrophy in the SSp and SSc continuously increase throughout adulthood, atrophy in the other RC muscles and the DM starts only from midlife onwards [17]. Furthermore, we found that torn RC muscles are characterized by an increase in extracellular matrix, and fatty droplets [17]. Despite high Pax-7 positive cells in torn muscles, their regeneration is limited [17]. Yet, the molecular mechanisms associated with these pathological conditions remain unknown.

Shoulder surgery provides a unique opportunity for ex-vivo investigations of functionally impaired muscles compared with unaffected muscles. Here we report a multidisciplinary study integrating radiological, histological, cellular and molecular investigations of diseased SSc and unaffected DM. We show that the SSc is highly degenerated and fibrotic but in contrast, viable muscle cell cultures were generated from both SSc and DM. Muscle cell cultures of SSc were predominantly indifferent from DM. However, reduced myogenesis in SSc cultures was associated with down-regulation of genes regulating muscle development as well as up-regulation of extracellular region genes.

## RESULTS

### Clinical characteristics

**Figure 1:**
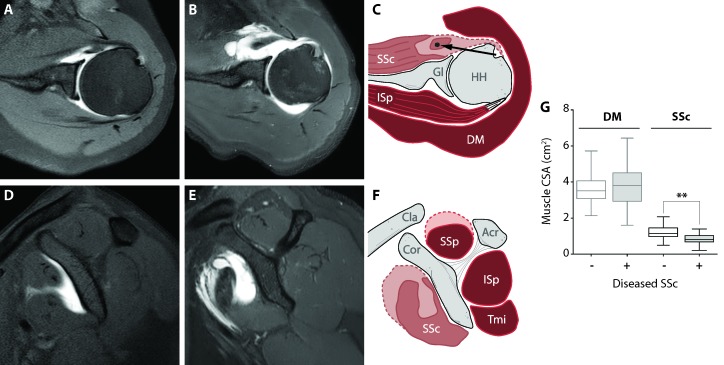
Clinical description of deltoid and subscapularis muscles Anatomical landmarks of shoulder muscles in intact and torn conditions. Representative MRA images from the control group (panels **A**. and **D**., transversal and coronal view, respectively) and from torn SSc muscle (panels **B**. and **E**., transversal and sagittal view, respectively). A schematic illustration of muscle cross-sectional surface area (CSA) is shown in panels **C**. and **F**. (transversal and coronal view, respectively). Muscle atrophy is indicated by the dashed line of the SSc. The long head of the biceps tendon is dislocated out of the bicipital groove, medially (black arrow). Subscapularis muscle (SSc), glenoid (Gl), humeral head (HH), infraspinatus (ISp), deltoid (DM), clavicle (cla), coracoid (cor), acromion (Acr), supraspinatus (SSp) and the teres minor (Tmi). Box plot (G) shows CSA of the DM and SSC between control subjects (n = 52) and patients with a diseased SSc (n = 28). Statistical significance between DM and SSc using unpaired t-test: ** *p* < 0.001.

Clinical features of diseased RC muscles were assessed from radiological imaging from 28 patients who underwent shoulder surgery. We compared RC muscle atrophy and fatty infiltration from patients with a diseased SSc with control subjects having an intact RC (Figure 1).

Patients with diseased SSc showed smaller cross-sectional area (CSA) of the SSc and SSp (28% and 32% lower, (*p* < 0.001 in both) respectively), whereas the RC muscles infraspinatus (ISp) and teres minor (Tmi), and the adjacent DM were not significantly different in size compared with subjects with an intact RC. In the patient group fatty infiltration was present in all four RC muscles, with highest fatty infiltration in the SSc. Among all four RC muscles, the largest contrast of atrophy and fatty infiltration between the patient group and control subjects was found in the SSc (Table 1). In the DM, no fatty infiltration was found and CSA was similar in both groups (Table 1). Therefore for ex-vivo analyses we compared SSc with DM.

**Table 1:** Clinical characteristics

Condition	Control subjects (*n* = 52)	Patients with diseased SSc (*n* = 28)	*p*-value
Age, years	46.0 (11.2)	58.7 (9.5)	**<0.001**
Female, N (%)	27 (52.9)	11 (39.3)	0.25
			
DM			
CSA, cm^2^	35.9 (7.9)	38.3 (11.6)	0.27
Fatty infiltration, N (%)	0 (0)	0 (0)	NA
SSc			
CSA, cm^2^	11.9 (3.1)	8.6 (2.8)	**<0.001**
Fatty infiltration, N (%)	0 (0)	22 (78.6)	**<0.001**
SSp			
CSA, cm^2^	5.0 (1.7)	3.4 (1.5)	**<0.001**
Fatty infiltration, N (%)	8 (15.7)	20 (71.4)	**<0.001**
ISp			
CSA, cm^2^	7.2 (1.8)	7.0 (2.3)	0.61
Fatty infiltration, N (%)	8 (15.7)	19 (67.9)	**<0.001**
Tmi			
CSA, cm^2^	3.7 (1.2)	3.6 (1.2)	0.68
Fatty infiltration, N (%)	0 (0)	6 (21.4)	**0.001**

Means (standard deviations) are provided unless otherwise stated. Deltoid (DM), cross sectional area (CSA), subscapularis (SSc), supraspinatus (SSp), infraspinatus (ISp), teres minor (Tmi). The p-values are calculated with unpaired t-tests, or chi-squared tests where appropriate.

### Muscle histopathology of the SSc and DM

**Figure 2:**
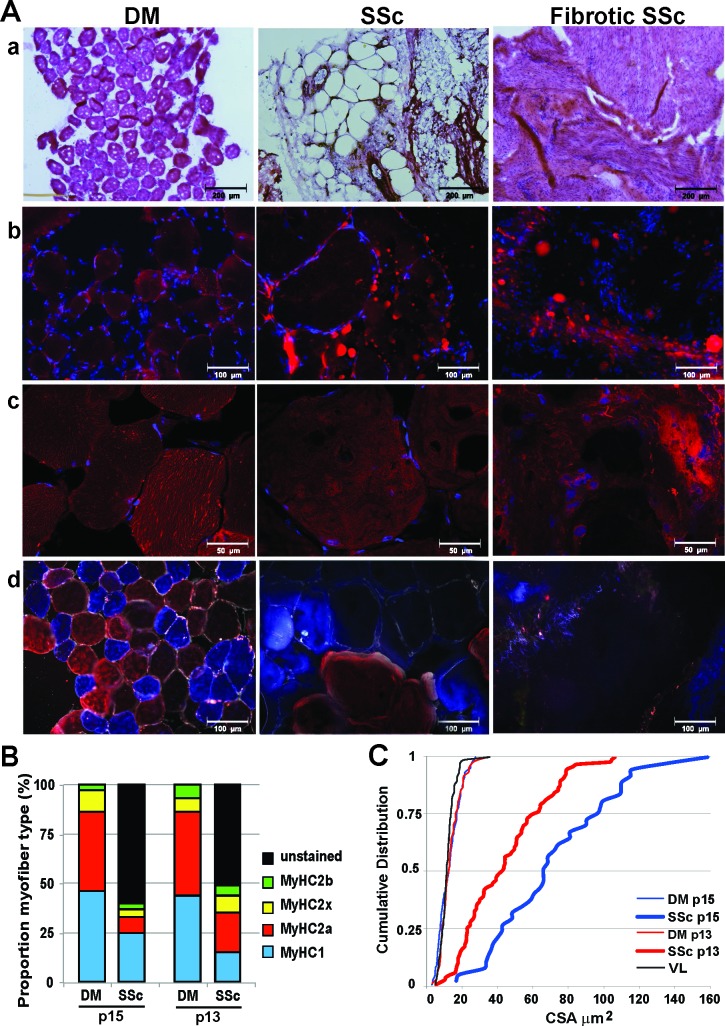
Histological analyses in deltoid and subscapularis muscles **A**. Representative images of deltoid (DM) and subscapularis muscle (SSc) stained with: a. H&E; b. Nile red (in red) for fatty droplets; c. C12-resazurin (in red) for oxidative metabolic activity. Nuclei are counterstained with DAPI in b and c ; d. Immunofluorescence with an antibody mix for MyHC-2b (green),-2a (red),-1 (blue) and laminin (white). For the SSc two conditions are shown: tissue containing myofibers (middle column) and highly fibrotic tissue without myofibers (right column). Images were taken with light microscope (a), fluorescence microscopes (b and d), or with a confocal microscope (c). Scale bars are 200 (a), 100 (b and d) or 50 (c) μm. **B**. and **C**. Analyses in paired samples for DM and SSc in two patients. B: Bar charts depict proportion of myofibers expressing MyHC-isotypes and those that were unstained. C: Cumulative distribution plot of the CSA in SSc, DM and vastus lateralis (VL) reference.

Muscle tissue was first investigated with H&E staining revealing that three out of five SSc samples were highly fibrotic and nearly no myofibers were present (Figure 2 and Table 2). In contrast, myofibers were found in all DM sections (Figure 2 and Table 2). Anti-laminin immunohistochemistry confirmed the presence of myofibers in all DM samples, however only 2 out of 5 SSc samples contained myofibers (Figure 2 and Table 2). Nile red staining showed the presence of large fatty droplets in the SSc, which were less common in the DM (Figure 2 and Table 2). This staining confirmed fatty infiltration found in radiological imaging.

**Table 2:** Histological observations in deltoid and subscapularis muscles

	Deltoid (*n* = 7)	Subscapularis (*n* = 5)
Myofibers in muscle	7/7	2/5
Fatty droplets	2/7	5/5
Fiber CSA, ∧μm^2^	62.1 (17.5)	105.8 (22.6)
MyHC-2b, ∧%	8.1 (6.9)	5.0 (3.7)
MyHC-2x, ∧%	9.6 (7.2)	7.0 (3.0)
MyHC-2a, ∧%	42.9 (13.8)	12.5 (10.6)
MyHC-1, ∧%	40.1 (12.2)	20.0 (7.1)
Unstained fibers, ∧%	0 (0)	55.0 (6.0)

Analysis per sample shows the number of positive samples out of total muscle samples. Analyses carried out only from myofiber-containing sections in the subscapularis are depicted with ∧. Fully degenerated muscles, as shown in Figure 2, were excluded from these analyses. Means (standard deviations) are provided unless otherwise stated. Cross sectional area (CSA), myosin heavy chain (MyHC).

Defects in metabolic activity, including glycogen catabolism, fatty acid oxidation, or mitochondrial oxidative phosphorylation often affect muscle function due to high energy consumptions [24]. In muscle wasting conditions oxidative metabolic activity can be changed [24]. Oxidative metabolic activity and oxidative phosphorylation can be monitored by an irreversible conversion of C12-resazurin to fluorescent resorufin, the fluorescent resorufin can record metabolic activity in the cell [25]. In undamaged deltoid muscles fluorescent foci of resorufin were scattered across the fibers, indicating active myofibers (Figure 2). However, in SSc muscles the distribution of fluorescent foci across myofibers was profoundly reduced (Figure 2). This indicates that oxidative metabolic activity and oxidative phosphorylation in the SSc are reduced.

We further investigated muscle pathology using the expression of myosin heavy chain (MyHC) isotypes. We applied an immunohistochemistry procedure with a mix of antibodies to MyHC-2b, -2a and -1 isotypes to record fiber type distribution [26, 27]. In both muscle tissues we found MyHC type-2a and type-1, but less frequent expression of MyHC-2b (Figure 2 and Table 2). In this immunostaining procedure non-stained fibers are regarded as MyHC-2x. To assess unstained myofibers, we then added an antibody to MyHC-2x. In DM samples all myofibers expressed one of the four MyHC isotypes (Figure S3), of which over 80% of the myofibers expressed either MyHC-2a or MyHC-1 (Table 2). In the SSc muscles, however, over 50% did not stain for any MyHC isotype. Of the myofibers that expressed MyHC, the majority expressed MyHC-1 (Table 2). Histological differences between diseased SSc and intact DM were further revealed in a paired analysis in the two patients with myofibers in the SSc. Above 50% of myofibers lost MyHC expression (Figure 2B) and myofiber size was dramatically increased compared with DM in the same patient (Figure 2C).

### Cellular analyses of SSc- and DM-derived myoblasts

**Figure 3:**
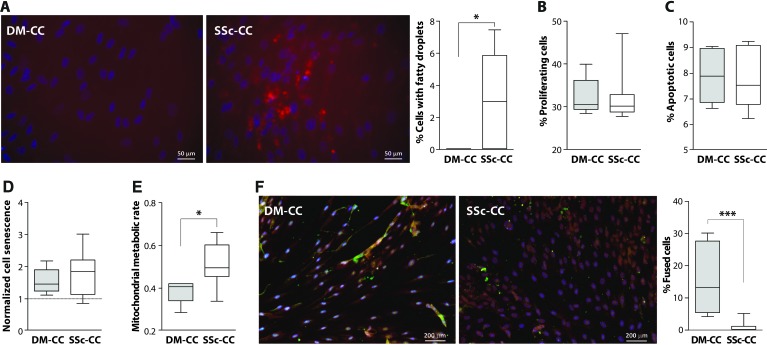
Cellular activities in deltoid (DM) and subscapularis (SSc) -derived muscle cell culture (CC) **A**. Nile red staining in deltoid (DM) and subscapularis muscle (SSc) cell culture (CC), nuclei are counterstained with DAPI (blue). Bar chart shows the percentage of cells containing fatty droplets. **B**. Bar chart shows the percentage of proliferating cells. **C**. Box plot shows the percentage of apoptotic cells. **D**. Box plot shows cell senescence (normalized to the reference vastus lateralis cell culture). **E**. Box plot shows the mitochondrial metabolic rate (normalized to the reference vastus lateralis cell culture). **F**. Representative images of fused cell cultures. Myoblasts are stained with Desmin (red) and fused cells are stained with MyHC (green). Nuclei are counterstained with DAPI (blue). Box plot shows the percentage of fused cells. Averages and standard deviations are from n = 5 (DM-CC) and n = 8 (SSc-CC). Statistical significance between DM-CC and SSc-CC using unpaired t-tests: * *p* < 0.05, *** *p* < 0.001.

To uncover the intrinsic molecular and cellular mechanisms underlying pathophysiological changes in chronic RC muscles, we generated muscle cell cultures from SSc muscles and Del as control. We applied this approach as the histology of chronically degenerated SSc muscle indicated that the tissue is highly fibrotic. Fatty droplets were prevalent in the SSc muscle cell cultures but were absent in the DM muscle cell cultures (Figure 3A). Within the desmin-positive cell population, myoblasts from the SSc and DM muscle cell cultures did not differ with respect to cell proliferation, apoptosis or cell senescence (Figure 3B-3E). The mitochondrial metabolic rate of the SSc myoblast cell cultures was higher than the DM myoblast cultures (Figure 3F). Moreover, we found that cell fusion in SSc derived cell culture was significantly lower compared with DM cultures (Figure 3G).

### RNA expression profiles in myoblasts

**Figure 4:**
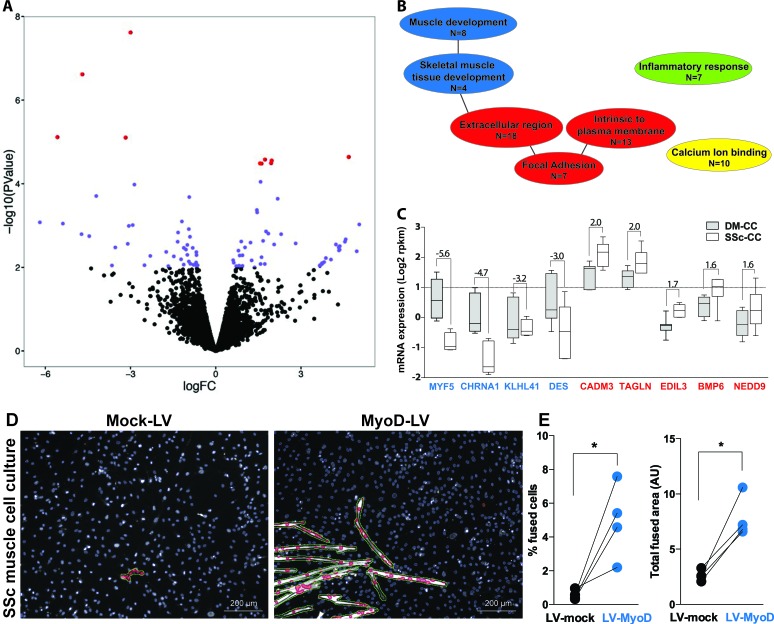
RNA expression profiles in deltoid and subscapularis muscle-derived cell cultures **A**. Volcano plot shows log2 fold change (FC) versus -log10 of the p-value of all genes found in the RNA-seq (pair-wise analyses, n = 7). Red dots indicate significantly dysregulated genes (*p* < 0.05 FDR). Blue dots indicate dysregulated genes with a nominal *p*-value < 0.01. Positive or negative FC indicates higher or lower expression in subscapularis (SSc) compared with the deltoid (DM) cell cultures (CC). **B**. Functional gene ontology (GO) of dysregulated genes (*p* < 0.01), daughter GO clusters are connected with a line. GO clusters of muscle development are depicted in blue, in red the extracellular region, in green the inflammatory system and in yellow calcium binding. N indicates the number of genes. **C**. Box plot shows expression levels in SSc-CC or DM-CC for the most significantly dysregulated genes (*p* < 0.05 FDR). Fold changes between DM and SSc are depicted. Genes of the GO muscle development are marked in blue and those of the extracellular region in red. **D**. Representative images of fused SSc cell cultures transduced with either mock or MyoD lentivirus (LV). Myoblasts are stained with MyHC (white) and segmented in green. Nuclei are counterstained with DAPI (blue) and segmented in blue. Nuclei within segmented MyHC objects are marked in red. Scale bar is 200 μm. **E**. Plots show paired analyses of % of fused cells and total fused area between mock and MyoD LV in 4 SSc cell cultures. *: *p*-value < 0.05, using a paired T-test.

Next we assessed molecular differences between the SSc and DM derived muscle cell cultures using RNA-seq in seven patients. A paired analysis of RNA-seq data from the two muscles of the same patient revealed only few molecular differences between the SSc and DM myoblasts (Figure 4A). Ultimately, using a false discovery rate (FDR) of 0.05, only 14 genes were identified as differentially expressed between the SSAC- and DM- derived cultures (114 with nominal *p*-value < 0.01) (Figure 4A). The 114 dysregulated genes were clustered into four functional gene-ontology groups: the extracellular region (*N* = 18) and focal adhesion (*N* = 7), calcium ion binding (*N* = 10), inflammatory response (*N* = 7) and four genes clustered in skeletal muscle development (Figure 4B). The most significantly dysregulated genes (*p* < 0.05 FDR) affecting muscle cell development were down-regulated, while genes within the extracellular region, were up-regulated in the SSc myoblast cultures (Figure 4C). The expression level of muscle stem cells genes did not differ between these two myoblast cultures (Figure S4).

To confirm that reduced expression of myogenic genes causes reduced cell fusion in SSc myoblasts cultures, we elevated MyoD expression levels using lentivirus transduction into SSc cultures, and mock virus was used as control. We assessed the myogenesis using expression of MyHC. We found that overexpression of MyoD restored myogenesis in the SSc cultures (Figure 4D). In average myogenesis was restored with an increase of 4.2% of fused cells (*p* < 0.05, paired T-test) and a 3.1 fold larger fused area (*p* < 0.05, paired T-test) (Figure 4E).

## DISCUSSION

Chronic muscle degeneration is highly prevalent in the elderly, characterized by loss of muscle mass and muscle strength, and leads to immobility and abrogates functionality and independence. In contrast to muscle waste in acute conditions, such as muscle disuse in denervation conditions [28] or starvation stress [29], the molecular mechanisms that are affected in chronic conditions are poorly understood. We present a multidisciplinary study revealing the most affected molecular mechanisms in degenerated muscles of chronic conditions, focusing on shoulder muscles. We chose to study shoulder muscles as the mechanical stresses on muscles around this joint are comparable. Furthermore, during shoulder surgery we could obtain two muscles, diseased and healthy, from each patient and those were paired analyzed.

We show that diseased SSc muscles are loaded with fatty infiltration, fibrosis, low metabolic activity, and reduced expression of MyHC isotypes. This is in agreement with our recent study showing that torn ISp muscle show similar histological marks including fatty infiltration, fibrosis and reduced expression of MyHC isotypes [17]. Reduced oxidative metabolism can suggest impaired mitochondrial activity [25], which is often found in degenerated muscles [24]. Moreover, in torn ISp we found a profound increase in collagen I staining, marking thickening of the extracellular matrix. This observation agrees with up-regulation of the extracellular matrix functional group we report here in torn SSc. Extracellular thickening is among the histological marks of chronic muscle degeneration and fibrosis [17, 30, 31]. In addition, we found that genes of the muscle functional group, specifically myogenesis genes, are down-regulated in cell culture from SSc compared with DM cell culture. This observation is in agreement with reduced myogenesis in the SSc cell culture. Moreover, overexpression of MyoD in SSC culture, which was found to be down-regulated, caused an increase in cell fusion. Restoring levels of additional genes from the muscle development group, and supressing expression of ECM genes could potentially lead to enhance muscle regeneration in chronic conditions.

Despite the pronounced histological changes in diseased SSc muscle, generation of myoblast cell culture from the diseased SSc was as successful as from the DM. Myoblasts cultures from degenerated SSc differed from DM cultures by only limited cellular and molecular signatures. We found no differences in cell proliferation, senescence and apoptosis between cell cultures from diseased SSc and DM. This observation is in agreement with a recent study showing no differences in cell proliferation between SSp and DM muscles [32]. In the torn SSp cell fusion was unaffected [32]. We, however, found reduced myogenesis in the torn SSc. This difference can be explained by differences in RC pathology between these two studies. A tear limited to the SSp is considered less severe RC disease, compared to tears also involving the SSc [8, 9, 33]. We therefore suggest that in more severe RC disease, muscle pathology worsens and cells have reduced myogenic capacity.

In chronically diseased SSc or ISp muscles [17] reduced expression of MyHC isotypes was complemented with an increase in CSA of myofibers. Genetic loss of MyHC expression causes an increased myofiber CSA [34]. This suggests that in chronically degenerated muscles reduced expression of MyHC isotypes could cause loss of myofibers construction and hence muscle weakness. It remains to be investigated how torn muscle conditions lead to loss of MyHC expression. In contrast to the chronic conditions investigated here, in animal models for RC tear that were generated by acute conditions (e.g. denervation, chemically, mechanically) MyHC expression is unaffected [35-40]. This suggests that molecular mechanisms associated with acute tear conditions differ from those in chronically diseased muscles.

Our results suggest that in chronically diseased SSc, muscle regeneration could be impaired due to reduced myogenesis of the muscle stem cells in an environment of thickened extracellular matrix. Targeting the extracellular region and muscle development could open new options for therapeutic developments such as cell therapies to enhance muscle regeneration. Repression of the extracellular region could be combined with surgical repair. Transplantation of *in vitro* propagated myoblasts into the surgical site could support muscle regeneration towards cell based therapy development. Cell therapy could ultimately enhance muscle regeneration in a combinatorial treatment to prevent muscle re-tear. Towards this, a recent study demonstrated grafting and fusion of satellite cells from non-degenerated muscles into injured muscles in mice [32]. In addition, efforts to reduce the extracellular region are developed in models for Duchenne muscular dystrophy [31], and those could be also applied for shoulder patients to order to regenerate myofibers.

This is an explorative study, with a small patient group and data have to be interpreted with caution. However, paired analyses increase statistical power and thus led to significant results. An independent replication study to validate our results should be performed. In this study we used muscle samples from shoulder pathology at advanced stage. In this stage the SSp is mostly torn, therefore the results presented here are an underestimation of muscle degeneration affecting only the SSp, which is more prevalent but less severe compared to tears extending to the SSc [8, 9, 33]. The more severe SSc pathology gives a broad spectrum to evaluate pathophysiological changes in chronic and torn muscles.

## MATERIALS AND METHODS

In this explorative study redundant muscle samples were collected as waste material from eleven patients that underwent shoulder surgery at the Leiden University Medical Center between 2012 and 2013 for *ex-vivo* analyses. All muscles samples were collected according to the Medical Ethical regulations of the institutional ethic review board of the Leiden University Medical Center and according to the guideline ‘good use of redundant tissue for clinical research’ by the Dutch Federation of Medical Research Societies. Anonymous use of redundant tissues as waste material for research purposes is according to a standard agreement with patients in our hospital.

### Surgical procedure and muscle collection

For shoulder surgery, patients were positioned in a beach chair pose and prepared and draped in a sterile fashion for shoulder surgery under general anesthesia. All patients received standard antibiotic prophylaxis before surgery. Due to damage of the SSc muscle, waste material for sampling could easily be obtained. In all cases the diseased SSc muscle was detached from the insertion site on the greater tubercle. Additionally, DM muscle samples (about 2mm^3^) were collected, which resulted from splitting the DM and pectoral muscle for the surgical approach. One muscle sample of each muscle was immediately frozen in liquid nitrogen and was stored at −80°C prior to the histological analyses, and the second sample was dipped into 100% Fetal Calf Serum (FCS) + 50μg/ml gentamycin for the establishment of myoblast cell cultures. The myoblast cell cultures were generated as described in [41] after 7-10 day *in vitro* propagation cultures reached 80-95% confluence in a 6 well plate and cells were kept frozen in liquid nitrogen.

### Histology and imaging

From seven DM and five SSc frozen samples were cryosectioned (20 μm) on dry ice using the Leica CM3050-S cryosection (Leica, Solms, Germany). Other samples were too small for cryosectioning. Histological analyses were: 1- hematoxylin and eosin (H&E) staining (Standard Operating Procedure: MDC1A_M.1.2.004). 2- myofiber typing protocol is detailed in Supplementary Methods file. 3- Fatty droplets staining with 1 μM Nile red (Sigma-Aldrich, Saint Louis, Missouri, USA) followed by PBS washing. Nuclei were counterstained with DAPI. 4- Oxidative metabolic activity was assessed with 10mM C12-Resazurin (Invitrogen) incubated for 15 minutes and followed by extensive washes with 50% methanol in PBS.

All slides were stained at the same day with the same mix. Slides were mounted with Polymount (Polyscience). C12-Resazurin fluorescence was visualized with a TCS-SP5 confocal microscope. Visualization of H&E stained sections was carried out with a Leica DFC550 light microscope. Fluorescent signals, otherwise, were captured with DM5500 microscope (Leica) using LAS AF software V2.3.6. or with Array scan VTI HCA (Thermofisher, Waltham, Massachusetts, USA). For each muscle, an unstained section was used to determine auto-fluorescence. Vastus lateralis (VL) cross-sections were used as a reference for each procedure and standardization of imaging. Per fluorophore, all muscle sections were imaged with the same configuration and exposure at the same day. Image overlay and image processing was carried out with Leica LAS AF version 2.6.0 build 7266. Image quantification was carried out with ImageJ (http://rsbweb.nih.gov).

### Analyses of myoblast cell cultures

For cellular and molecular analyses myoblast cell cultures were seeded simultaneously. Cellular analyses were carried out on cultures at passage 1 from eight SSc samples and five DM samples that reached 20,000 cells at the same time. Muscle cell fusion and cell senescence were carried out on cells at passage 2. Cellular analyses are schematically summarized in Supplementary Figure S1. RNA-seq was carried out on seven paired cultures at passage 2. The researchers were blinded to the muscle type during experimentation and analyses. The cellular analyses were performed as summarized and detailed in the Supplementary Methods file.

### RNA isolation and RNA analyses

Cultures from seven pairs were included in the final analysis after passing control quality (Supplementary Table S1). RNA was isolated from cultures at passage 2 grown in a 12 well plate using the Nucleospin RNA II (Macherey-Nagel, Düren, Germany) according to the instructions of the manufacturer, and was stored at −80°C prior to analyses. RNA quality was assessed using the Agilent 2100 Bioanalyzer (Agilent Technologies, Santa Clara, California, USA). RNAseq library preparation and pair-wise analysis to identify SSc dysregulated genes is detailed in the Supplementary Methods file.

### Lentiviral transduction

4 cultures of SSc at passage 2 were transduced with MyoD lentivirus [42] or control lentivirus (multiplicity of infection (MOI): 5. After 3 days cells were incubated at fusion conditions, 2% horse-serum in DMEM, for 5 days. Quantification of fused cells was carried out as detailed in the Supplementary Methods file. An average of 9158 nuclei per condition was included.

### Radiological imaging

Due to medical ethical constrains we were not allowed to associate muscle samples with medical information, therefore all MRIs of patients undergoing shoulder surgery involving SSc muscle damage (*n* = 28) between 2012 and 2013 were evaluated. Patients received local intra-articular analgesia and a contrast agent for arthrography *via* a posterior approach. Magnetic Resonance imaging with arthrography (MRA) scanning and analyses were performed as previously detailed [9, 17]. As control group, 52 subjects from a previous study were included with structurally intact RC muscles [9]. The MRI scan of one control could not be reviewed. The institutional ethics review board waived the need for participant informed consent to review the MRIs. The MRIs were anonymized and observers were blinded for outcomes.

### Statistical analyses

All statistical analyses were performed using IBM SPSS statistics version 20 (IBM Corp, 2011, Armonk, New York, USA). Categorical data are expressed using absolute values and percentages. Parametric continuous data are described with means and 95%-confidence intervals (95%-CI). Statistical assessments were carried out with unpaired Student's t-tests, unless otherwise stated.

## SUPPLEMENTARY MATERIALS FIGURES AND TABLES


