# Supplementary material for: Molecular signatures of age-associated chronic degeneration of shoulder muscles
Source: Oncotarget. 2016 Feb 14;7(8):8513–23. doi: 10.18632/oncotarget.7382 (PMC4890983; doi:10.18632/oncotarget.7382)
Supplement: Supplementary file 1 [file oncotarget-07-8513-s001.pdf]

Molecular signatures of age-associated chronic degeneration of the shoulder muscles

Supplementary Material

Methods

Histology

Immunostaining for Myosin heavy chain isotypes was carried out as described in[1], with the following modifications: non-fixed cryosections were directly incubated with a primary antibody to Laminin (1:1000; ab11575, Abcam, Cambridge, UK). When indicated, a supernatant of hybridoma 6H1 (1:5, Developmental Studies Hybridoma Bank (DSHB), National Institutes of Health, Iowa City, Iowa, USA), antibody to MyHC--2x was co-incubated. These antibodies were detected with anti-rabbit-Alexa-647 conjugated and anti-mouse-Alexa-564 (Molecular Probes, Invitrogen, Waltham, Massachusetts, USA). After extensive washings the sections were co-incubated with a mixture of monoclonal antibodies to MyHC type-1, type-2a and type-2b proteins (hybridoma BA-D5, SC-71 and BF-F3, respectively (DSHB)), conjugated with Alexa-350, Alexa-594 and Alexa-488, respectively. Antibody production from hybridomas and fluorophore conjugation were carried out as described in[2]. All antibody incubations and washes were carried out in PBS containing 0.05% tween and 5% dry milk. Slides were mounted with Aqua Polymount (Polyscience, Warrington, Pennsylvania, USA).

Cell culture and cellular analyses

All cells were seeded in a 12 well plate and an assay was carried out on cultures at the same passage number, only cultures that grew to confluence at each passage were included in the analyses. In addition a myoblast cell culture from human Vastus lateralis was used as a reference for cellular measurements. An immunostaining to Desmin was used to determine the proportion of myoblasts in each cell culture (Supplementary Figure 3). Using the LSR-II flow cytometer (BD, East Rutherford, New Jersey, USA) we estimated the proportion on Desmin positive cells to be ~80% in average in the myoblast cell culture, which is similar from the VL cell culture Figure S2B. Analyses were carried out on the Desmin positive cell population.

Analyses of cell proliferation, apoptosis, mitochondrial rate activity and fatty droplets staining were all performed in cell cultures at passage 1. Analyses of cell senescence, cell fusion, and RNA extraction for molecular studies were performed on cultures at passage 2. Cell proliferation was carried out with the Click-iT EdU Pacific Blue Kit (Life Technologies, Carlsbad, California, USA). Cells were incubated with 1µM EdU for 4 hours and further according to the manufacturer instructions. Subsequently cultures were incubated with Annexin V Apoptosis Kit-PE (ApoScreen, Southern Biotech, Birmingham, Alabama, USA) according to the manufacturer instructions, followed by incubation with an anti-Desmin-Cy7-congujated antibody (Stratech, Newmarket, UK). For analysis of the mitochondrial rate activity cultures were labeled with 1 µM JC-1 (Invitrogen) for 20 minutes, followed by staining with an anti-Desmin antibody. Senescent cells were detected with beta-galactosidase as described in[3]. Fluorescence signals were analyzed with the LSR-II flow cytometer (BD) as follows: cells were first gated on size (FSC/SSC) and subsequently on desmin signal. Only desmin positive cells (~50,000 per analysis) were included in the subsequent analyses. In all experiments a myoblast cell culture from human vastus lateralis (VL) muscle was included as a reference for the procedure, since the VL muscle is used in all sarcopenia research[4]. Using the FACSDiva software (BD) we calculated the proportion of EdU positive cells (proliferation), AnnV positive cells (apoptosis), the mitochondrial metabolic rate (MFI for the PE, divided by the MFI for the FITC channels), or the MFI for beta-galactosidase (cell senescence). All values were standardized to the VL control.

Intracellular lipid droplets were detected with 1 µM NileRed (Sigma-Aldrich) for 20 minutes. Cultures were washed and nuclei were counter stained with Hoechst prior to imaging. Nile red signal was imaged with the Arrayscan VTI HCA. For cell fusion, cells from passage 2 were seeded into a 48 well plates in triplicates, and fusion was carried out in 2% horse-serum for 7 days. The detection of fused cells was carried out in fixed and permeabilized cell cultures[5]. Staining with MF20 (mouse-anti-MyHC antibody, Sigma-Aldrich) and secondary anti-mouse-Alexa488-conjugated marks fused cells, and rabbit-anti-Desmin antibody (AbCam) and secondary anti-rabbit-Alexa 594 marks myoblasts.

Prior to imaging with Array scan VTI HCA cell nuclei were counter stained with Hoechst[5]. Imaging with Array scan VTI HCA was carried out as follows: Nile---Red was imaged with a Cy5 filter and Hoechst with a DAPI filter. MF20 and Desmin were images with a FITC and TexasRed filter, respectively. Image quantification was carried out using ArrayScan VTI HCA software.

RNAseq

RNAseq library preparation was carried out as described in[6]. Strand specific RNAseq libraries were generated using the method described by Parkhomchuk et al. with minor modifications[6]. In short, mRNA was isolated from 1 µg total RNA using oligo-dT Dynabeads (61002, Life Technologies, Carlsbad, California, USA) and fragmented to 150-200 nt in first strand buffer for 3 minutes at 94 C. Random hexamer primed first strand was generated in presence of dATP, dGTP, dCTP and cTTP. Second strand was generated using dUTP instead of dTTP to tag the second strand. Subsequent steps to generate the sequencing libraries were performed with the NEB Library prep mastermix kit (E6040L, New England Biolabs, Ipswich, Massachusetts, USA) for Illumina sequencing with minor modifications, i.e., after indexed adapter ligation to the dsDNA fragments, the library was treated with USER enzyme (M5505L, New England Biolabs) in order to digest the second strand derived fragments. After amplification of the libraries, samples with unique sample indexes were pooled and paired-end 2x125 bp sequenced on a single HiSeq2000 lane (Illumina, San Diego, California, USA) with 2x90-nucleotide-long Paired End reads, resulting in a minimum of 3 Gb clean data per sample Table S2. Sequencing analysis was performed using in-house pipeline Gentrap, the source code is available at (https://git.lumc.nl/rig-framework/gentrap), including sequencing quality evaluation using fastqc toolkit (version 0.10.1) (http://www.bioinformatics.babraham.ac.uk/projects/fastqc/), reads clean-up (adapter clipping followed by quality trimming) and then alignment of reads to the Human reference genome build 19 (hg19/GRCh37) using GSNAP (version 2013-10-12)[7]. RNA-seq quality from individual samples is shown in Figure S3. The alignment quality was assessed using Picard (v1.86)

(http://broadinstitute.github.io/picard/). On average, 95% of cleaned reads were mapped, and 94% of mapped reads were in exon regions (Table S2). Differential RNA expression analysis of genes from the SSc and DM derived myoblast cell cultures were compared using a paired analysis. Expression level was calculated based on Ensembl transcripts (downloaded on 2014-08-08 from UCSC genome browser) using HTSeq[8;9]. The normalization and statistical testing was performed using EdgeR version 3.6.8 (Bioconductor, Seattle, Washington, USA) as previously described[10]. Gene expressions were normalized with the Trimmed Mean of M-values (TMM). Genes with mean read per kilo-base per million (RPKM) greater than 1 were then selected for statistical testing. The maximum likelihood ratio test was performed as previously described to identify pairwise differentially expressed genes using the generalized linear model (GLM)[9]. Genes with p-value ≤ 0.05 false discovery rate (FDR) were selected as differentially expressed. Clustering of differential expressed genes to functional groups was performed in DAVID clustering using gene-ontology[11]. RT-qPCR for 7 genes was carried out in order to assess RNA quality (Table S2A).

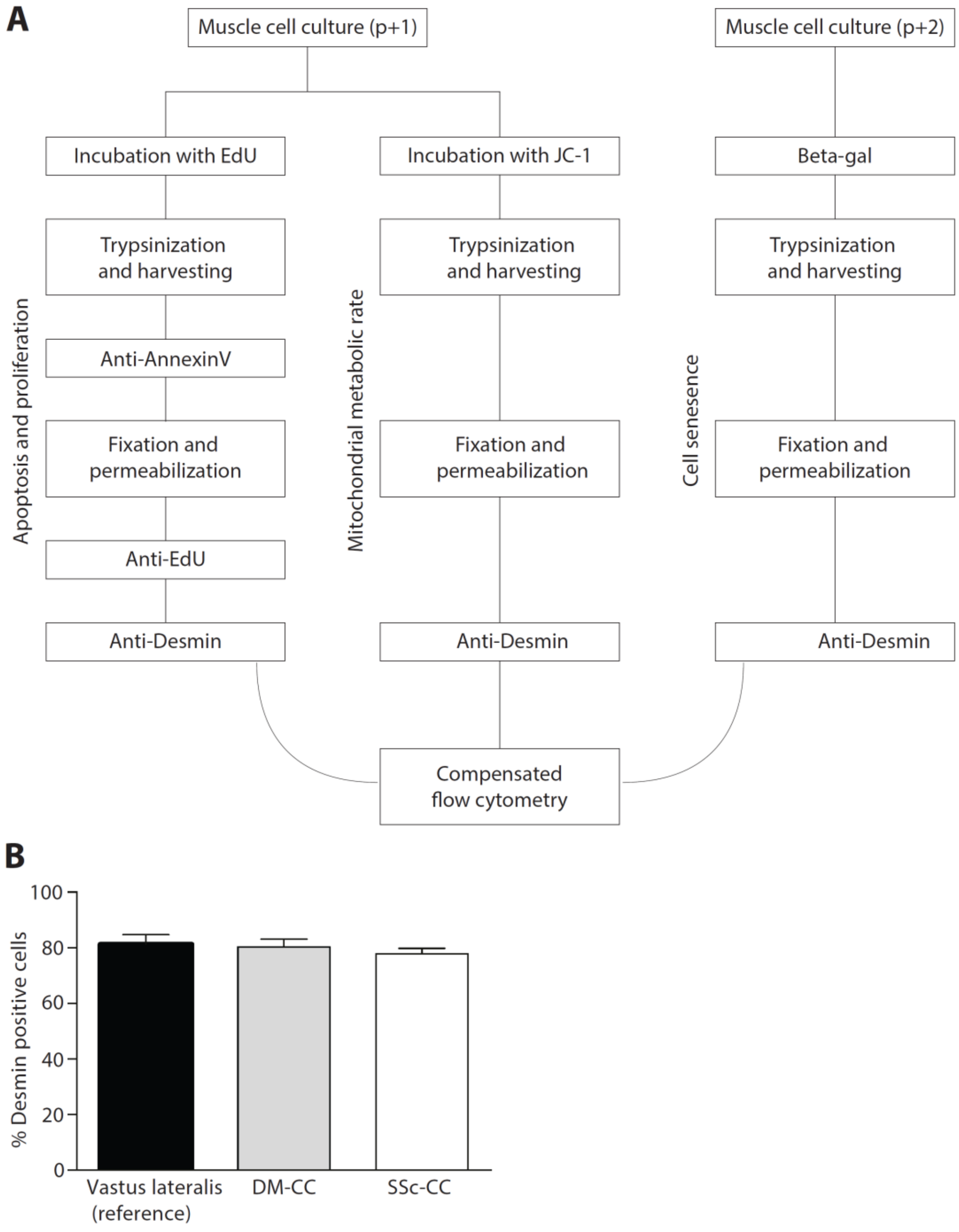

Figure S1: Flow-cytometry analysis of muscle cells cultures

(A) Flowchart shows a summary of the experimental procedures and analysis of muscle cells cultures with the flow cytometer.

(B) Bar chart shows the mean fraction of Desmin positive cells in the DM, SSc and vastus lateralis (reference) Cell Culture (CC)

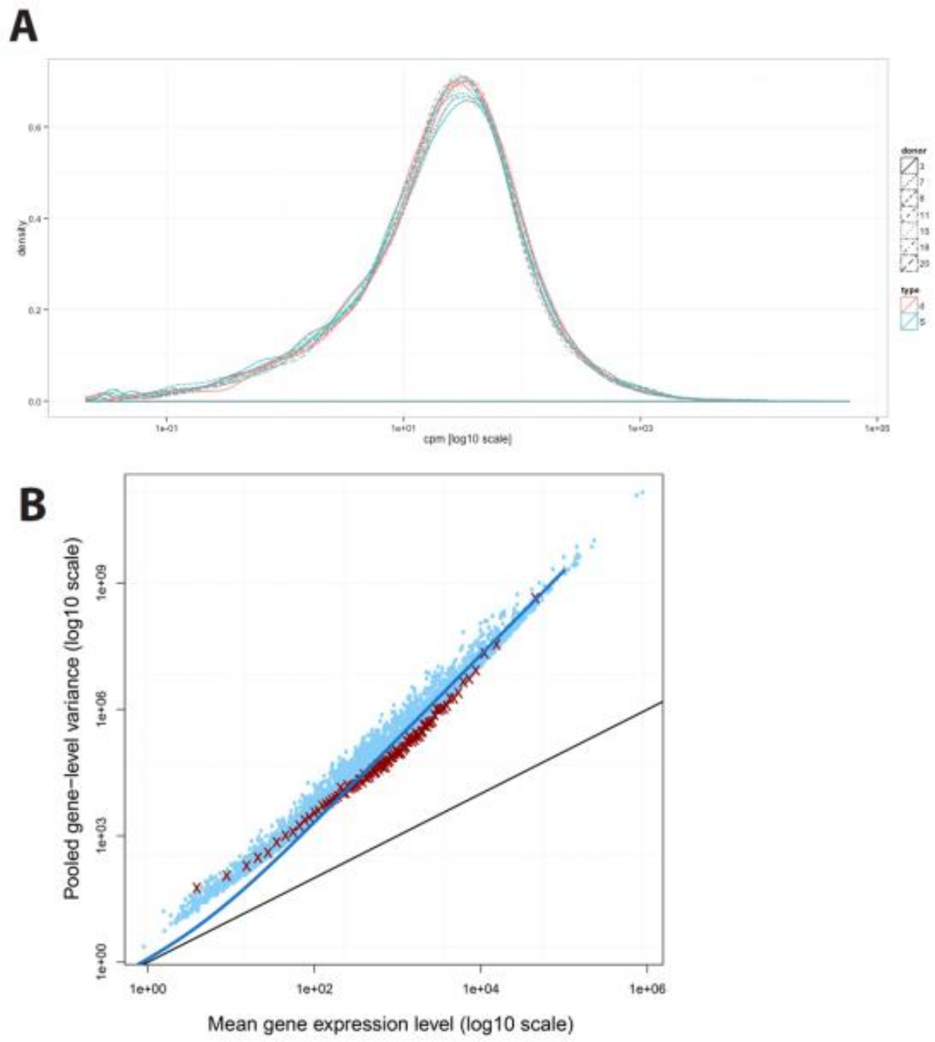

Figure S2: RNA-seq quality control

(A) Plot shows the distribution of normalized counts (cpm) and density per sample.

(B) Light blue circles show the estimated genewise/tagwise variances on the mean-variance plot. Dark red crosses show the average of the raw variances for each bin of tags plotted against the average expression level of the tags in the bin. Averages are taken on the square root scale as regular arithmetic means are likely to be upwardly biased for count data, whereas averaging on the square scale gives a better summary of the mean-variance relationship in the data. Blue curved line shows the mean-variance relationship for a NB model with common dispersion.

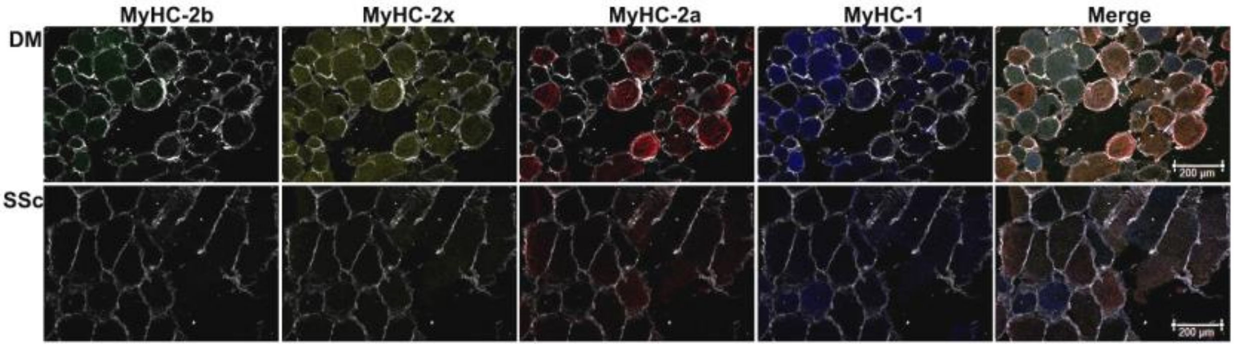

Figure S3: Myofiber-typing using five antibodies

Immunostaining was carried out with antibodies to four MyHC isotypes (-2b; -2x; -2a and -1). Myofiber surface is stained with anti-laminin (white). Images were taken with the Cellomics. Shown are representative muscle section, scale bar is 200µm.

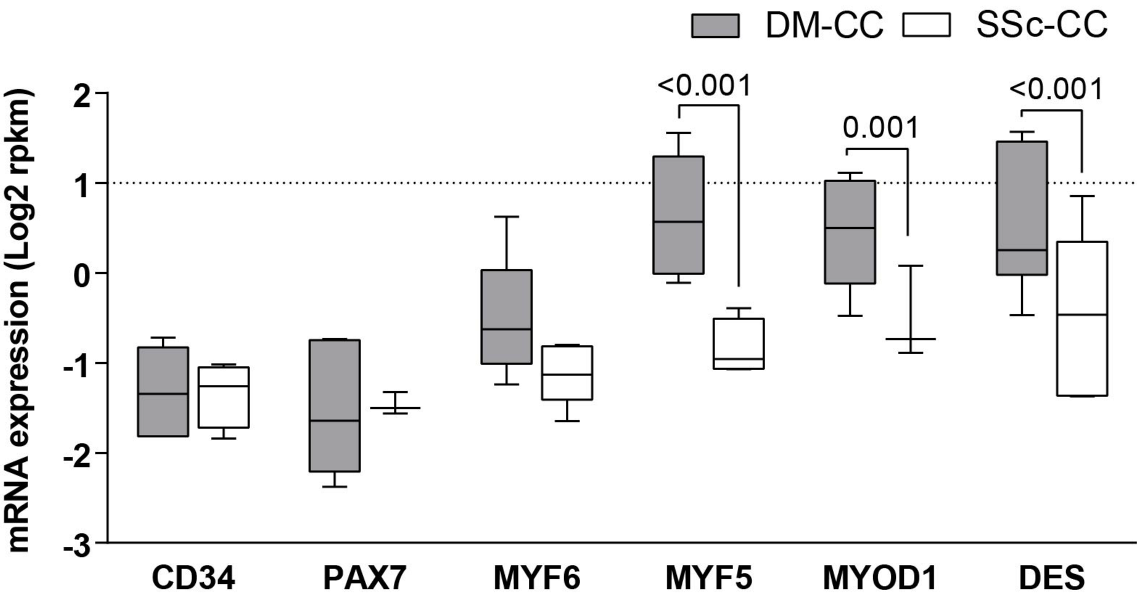

Figure S4: Assessment of myogenic gene expression from RNA-seq

Box plot shows the expression levels in DM or SSc derived Cell Culture (CC). Expression values (log2) were obtained from unfiltered cpm values. Averages and SD are from 7 samples.

Table 1: Summary of RNA-seq quality

id	Age	Gender	Tissue	Total reads	Mapped reads (%)	Mapped pair (%)	Spliced reads (%)	insert size	R1raw GC	R2raw GC	R1clean GC	R2clean GC	% mapped to exons
p15	43	M	DM	43025664	96,30	91,56	42,86	249	49	50	49	50	91,12
			SSc	37761171	94,11	89,45	41,42	227	48	50	48	49	95,32
p8	44	M	DM	44400673	95,50	94,65	42,03	320	48	50	48	50	95,62
			SSc	44040460	91,98	91,09	35,60	342	47	48	47	48	89,33
p18	64	F	DM	59817071	97,86	97,37	43,70	258	48	49	48	49	96,25
			SSc	40803244	95,84	94,99	43,15	228	48	50	48	49	93,33
p11	68	F	DM	51736276	93,99	90,24	43,38	238	49	50	49	50	95,79
			SSc	47187673	97,36	93,94	45,49	228	50	51	49	51	96,33
p3	69	M	DM	28381564	96,09	92,64	43,25	207	49	50	49	50	95,84
			SSc	24976493	96,16	92,47	43,30	228	48	49	48	49	96,34
p7	75	F	DM	44197564	96,58	95,94	42,51	228	49	50	49	50	95,95
			SSc	37347223	97,40	96,77	41,90	258	48	49	48	49	95,73
p20	77	F	DM	48851259	87,53	86,96	38,68	238	48	49	48	49	94,92
			SSc	43938679	83,95	83,06	29,62	259	47	48	47	48	91,94

Cleaned reads: Reads remained after adapter clipping and quality trimming, with minimum length > 25. Mapped reads: Reads that were mapped to GRCh37/hg19 reference genome. Mapped pair: Both reads from the same fragment (paired end reads) were mapped to reference genome. Spliced reads: Reads spanning exon-exon junctions after mapping. R1: The first read in a paired end reads. R2: The second read in a paired end reads.

Table S2: RNA

Table S2A: Expression correlation between RNA-seq and RT-qPCR for selected genes.

paired t-test per patient		paired t-test per gene	
p3	0,984	GUSB	0,863
p7	0,132	PABPN1	0,126
p8	0,534	FOXO3	0,226
p11	0,593	MURF1	0,339
p15	0,512	SIRT1	0,380
p18	0,547	CDKN1A	0,477
p20	0,042	PPARg	0,391

RT-qPCR was carried out with specific primers (Table S2B) for the seven listed genes in the 7 samples in triplicates. Fold changes between subscapularis (SSc)-Cell Cultures (CC) and deltoid (DM)-CC were calculated from expression values. To validate RNA-seq RT-qPCR was performed on 7 selected genes and a correlation between RT-qPCR and RNA-seq fold changes between RNA expression in DM and SSc samples was assessed using a paired t-test per patient or per gene. For all seven genes p-value was insignificant, indicating that the fold changes between RNA-seq and RT-qPCR do not differ.

We selected FOXO3, MURF1, SIRT1, PPARg, CDKN1A and PABPN1 for this as all were reported as aging-associated in skeletal muscles[12]. Primers for RT-qPCR are listed in Table S2B. RT-qPCR was performed using 3ng template and Cyber Green (Invitrogen). Fold changes were calculated according to the DDCT method, after normalization to the mean of *GUSB* and GAPDH housekeeping genes and DM samples.

Table S2B: Primer list for genes used for RT-qPCR analysis

	Forward primer	Reverse primer
GUSB	CTCATTTGGAATTTTGCCGATT	CCGAGTGAAGATCCCCTTTTTA
MURF1	TTCATTGAAGAAGAAGATCAGGAA	CCTGGGCCTGTCACCAAG
SIRT1	GGACATGCCAGAGTCCAAGT	GCTGGTGGAACAATTCCTGT
CDKN1A	TATGGGGCTGGGAGTAGTTG	AGCCGAGAGAAAACAGTCCA
FOXO3	GATGCTGATGGGTTGGATTT	GAGAGCAGATTTGGCAAAGG
PPARg	TGAATGTGAAGCCCATTGAA	CTGCAGTAGCTGCACGTGTT
PABPN1	CGTTGGCAATGTGGACTATG	ACACGGTTGACTGAACCACA

References

(1) Schiaffino S, Reggiani C. Fiber types in mammalian skeletal muscles. *Physiol Rev* 2011;91:1447-1531.

(2) Klooster R, Straasheijm K, Shah B et al. Comprehensive expression analysis of FSHD candidate genes at the mRNA and protein level. *Eur J Hum Genet* 2009;17:1615-1624.

(3) Noppe G, Dekker P, de Koning---Treurniet C et al. Rapid flow cytometric method for measuring senescence associated beta---galactosidase activity in human fibroblasts. *Cytometry A* 2009;75:910-916.

(4) Righolt CH, van 't Hoff ML, Vermolen BJ, Young IT, Raz V. Robust nuclear lamina---based cell classification of aging and senescent cells. *Aging (Albany NY)* 2011;3:1192-1201.

(5) Greenspan P, Mayer EP, Fowler SD. Nile red: a selective fluorescent stain for intracellular lipid droplets. *J Cell Biol* 1985;100:965-973.

(6) Parkhomchuk D, Borodina T, Amstislavskiy V et al. Transcriptome analysis by strand-specific sequencing of complementary DNA. *Nucleic Acids Res* 2009;37:e123.

(7) Wu TD, Nacu S. Fast and SNP-tolerant detection of complex variants and splicing in short reads. *Bioinformatics* 2010;26:873-881.

(8) Anders S, Pyl PT, Huber W. HTSeq-a Python framework to work with high-throughput sequencing data. *Bioinformatics* 2015;31:166-169.

(9) Robinson MD, McCarthy DJ, Smyth GK. edgeR: a Bioconductor package for differential expression analysis of digital gene expression data. *Bioinformatics* 2010;26:139-140.

(10) Robinson MD, Oshlack A. A scaling normalization method for differential expression analysis of RNA---seq data. *Genome Biol* 2010;11:R25.

(11) Bammler T, Beyer RP, Bhattacharya S et al. Standardizing global gene expression analysis between laboratories and across platforms. *Nat Methods* 2005;2:351-356.

(12) Anvar SY, Raz Y, Verway N et al. A decline in PABPN1 induces progressive muscle weakness in oculopharyngeal muscle dystrophy and in muscle aging. *Aging (Albany NY)* 2013;5:412-426.
